# A Piezoelectric Heterostructure Scavenging Mechanical Energy from Human Foot Strikes

**DOI:** 10.3390/mi13081353

**Published:** 2022-08-20

**Authors:** Wei He

**Affiliations:** School of Information Engineering, Baise University, Baise 533000, China; weiheky@yeah.net

**Keywords:** piezoelectric heterostructure, mechanical energy, human foot strike, magnetic spring, piezoelectric unimorph cantilever

## Abstract

This paper presents a piezoelectric heterostructure for extracting mechanical energy from human foot strikes based on the impact of a moving block on the tips of the piezoelectric unimorph cantilevers. The use of the magnetic springs allows low-frequency and high-amplitude movements of the device. The piezoelectric unimorph cantilevers deform under a human foot strike on the pedal, and the piezoelectric elements in *d*_31_-mode produce output voltages. An analysis was conducted, and the working principle was stated. A prototype was fabricated to validate the feasibility of the proposed design. The experimental results show that the generated RMS voltage increases with human walking (running) speed. At the walking speed of 6 km/h, an average power of 36.26 μW is produced across a matching resistive load of 4 MΩ with an initial separating distance of 9 mm. Improvements of the device are possible, allowing an increase in the average power by increasing the number of piezoelectric unimorph cantilevers and using the piezoelectric materials with higher piezoelectric constants.

## 1. Introduction

With the rapid development of wearable electronic devices, higher requirements for the power supplies of the devices have arisen. At present, these electronic devices are usually powered by batteries, but the available energy of the batteries is limited. Furthermore, frequent battery replacements will bring inconvenience to the applications of the electronic equipment. Researchers have put forward schemes to convert the energy originating from the temperature differences [1,2] sunlight [3], magnetic field [4,5,6], and mechanical vibration [7,8,9,10] into electrical energy to power the electronics, which is called energy-harvesting technology. Energy harvesting can effectively solve the power supply problem of low-power electronic equipment. For wearable electronic devices, there are great prospects for harvesting energy from the body as the human body can produce different forms of energy throughout daily life. Mechanical energy with high energy density produced by human motions (e.g., walking, running, jumping, arm swing) is easy to capture and can be readily found. Technologies for harvesting mechanical energy from human motions to supply power to low-power electronic devices have attracted growing attention, mainly including electromagnetic [11,12,13,14,15], piezoelectric [16,17,18,19], and magnetoelectric [20] methods.

A human foot strike exerts a large force on the insole, and the mechanical energy originating from the foot strikes can be harvested by inserting an energy-harvesting device into a shoe. Some researchers used triboelectric materials embedded in the insole to scavenge the mechanical energy produced by foot strikes [21,22]. The triboelectric generators usually occupy large areas in the shoes. Piezoelectric energy-harvesting devices have been developed for foot strikes [23,24,25]. An amplification mechanism was used to harvest energy from human footsteps [23]. Output powers can be generated with high efficiency due to the large mechanical strain. A piezoelectric sandwich structure was fabricated [24] that is compatible with a shoe for extracting energy from foot strikes. However, protection mechanisms were not devised for the piezoelectric materials of the devices reported in [23,24] under large pressure resulting from the foot strikes. Recently, a shoe-mounted piezoelectric harvester was presented [25], who explored the utilization of three different excitations produced by the foot. The harvester uses traditional springs to generate the recovery force. However, traditional springs might not be preferred under large pressure induced by foot strikes due to the intrinsic limitations of the mechanical springs. In this paper, an impact-based piezoelectric heterostructure using magnetic springs is developed for extracting energy from human foot strikes. The use of the magnetic springs makes the proposed device suited to the low-frequency (e.g., less than 2 Hz) and high-amplitude movements under large pressure resulting from foot strikes. A protection mechanism is used to protect the piezoelectric unimorph cantilevers, and the maximum tip displacement is adjustable (within the permitted impact load). The feasibility of the device was experimentally validated. The device exhibits a monotonous increase in the RMS voltage as the walking (running) speed increases from 3 km/h to 7 km/h. The generated maximum average power increases with the initial separating distance *d*, which reaches 36.26 μW for *d* = 9 mm at 6 km/h (the matching load resistance is 4 MΩ). Further optimization of the piezoelectric heterostructure is possible, which might allow for large increases in the induced RMS voltages and the average power.

## 2. Design and Analysis

**Figure 1:**
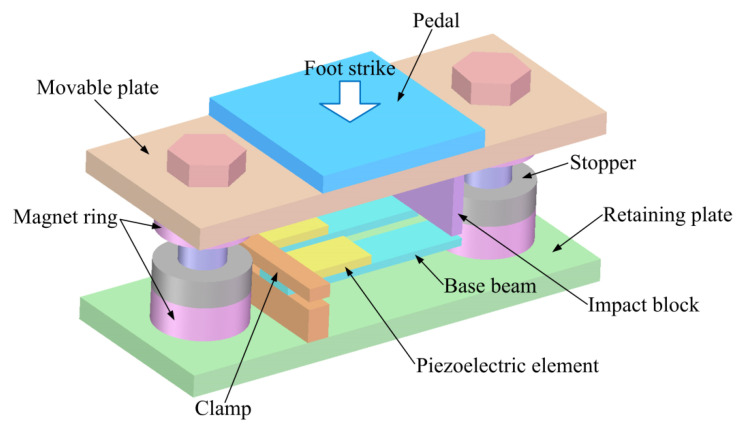
Schematic diagram of the proposed piezoelectric heterostructure with a protection mechanism.

Figure 1 shows the schematic diagram of the presented piezoelectric heterostructure. The heterostructure is composed of two piezoelectric unimorph cantilevers, two magnetic springs, a movable plate, a retaining plate, a pedal, an impact block, two stoppers, and the auxiliary components (e.g., screws, clamp, outer frame). Each piezoelectric unimorph cantilever is constructed from a base beam (26 mm× 8 mm× 0.8 mm for each base beam) and a piezoelectric element (12 mm × 8 mm × 0.6 mm for each piezoelectric element). The materials in the magnetic rings and the piezoelectric elements are, respectively, NdFeB and PZT5H. The internal and external diameters of each magnet ring are 10 mm and 18 mm, respectively. The movable plate, the retaining plate, the pedal, the impact block, the clamp, and the outer frame are made up of aluminum alloy (6061). The maximal dimension of the assembled prototype is 80 mm × 40 mm × 42 mm (respectively along the directions of length, width, and height) under operation. The movable part is constructed from the pedal, the movable plate, the impact block, and the top magnet rings, and a downward movement of the movable part will be induced when the pedal is struck. The impact block then strikes the tips of the based beams (the stoppers are used to protect the cantilevers and to prevent direct collisions of the magnetic rings), which causes the based beams to bend. The stress is transmitted to the piezoelectric elements, which produce output voltages due to the piezoelectric effect (*d*_31_ mode). The movable part then returns to its original position for the next gait cycle due to the repulse force of the magnetic springs.

**Figure 2:**
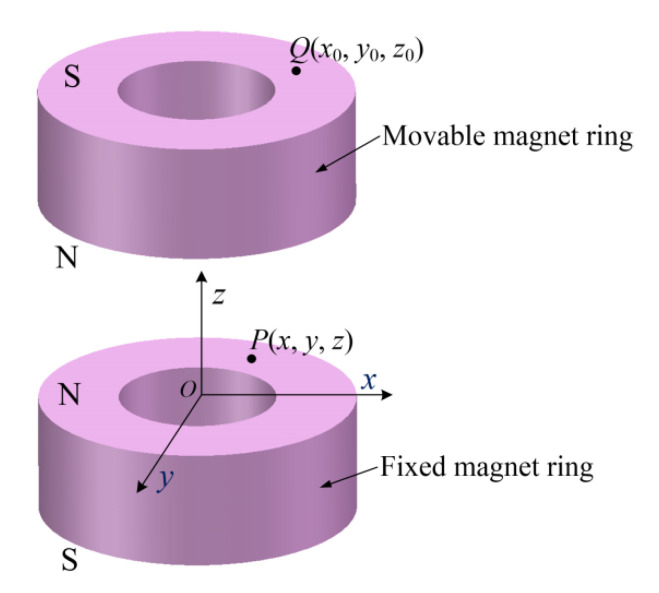
Schematic diagram of the magnetic spring.

Figure 2 shows the schematic diagram of one magnetic spring of the heterostructure. Based on the concept of magnetic charge, the positive magnetic charge is distributed on the N poles, and the negative magnetic charge is distributed on the *S* poles. The *z*-component magnet force *F_z_* on the movable magnet ring is of primary concern. The magnetic flux intensity at *Q*(*x*_0_, *y*_0_, *z*_0_) on the magnetic pole surface *S*_3_ (*S* pole of the movable magnet ring) induced by *P*(*x*, *y*, *z*) on the magnetic pole surface *S*_1_ (N pole of the fixed magnet ring that is fixed on the retaining plate) can be expressed as

(1) $$dB_{z}=\frac{\sigma_{m}dS_{1}}{4\pi {\left|r\right|}^{3}}(z_{0}-z),$$

where |*r*| is the distance between *P* and *Q*, $\left|r\right|=\sqrt{{\left(x_{0}-x\right)}^{2}+{\left(y_{0}-y\right)}^{2}+{\left(z_{0}-z\right)}^{2}}$, *σ_m_* is the surface magnetic charge density, and *σ_m_dS*_1_ represents the corresponding surface magnetic charge. Thus, the *z*-component magnetic flux intensity at *Q*(*x*_0_, *y*_0_, *z*_0_) produced by the fixed magnet ring can be expressed as

(2) $$B_{z}=\iint_{S_{1}}\frac{\sigma_{m}dS_{1}}{4\pi {\left|r\right|}^{3}}(z_{0}-z)-\iint_{S_{2}}\frac{\sigma_{m}dS_{2}}{4\pi {\left|r\right|}^{3}}(z_{0}-z),$$

where *S*_2_ is the magnetic pole surface (S pole of the fixed magnet ring) with a surface magnetic charge density of −*σ_m_*. For the surface magnetic charge −*σ_m_dS*_3_ on S_3_, the resulting *z*-component magnetic force induced by the fixed magnet ring is

(3) $$dF_{z}=-\sigma_{m}dS_{3}H_{z}.$$

where *H*_z_ is the magnetic field, *H_z_* = *B_z_*/*μ*_0_ and *μ*_0_ is the permeability of vacuum.

The *z*-component magnetic force on the movable magnet ring is calculated by

(4) $$F_{z}=-\iint_{S_{3}}\sigma_{m}H_{z}dS_{3}+\iint_{S_{4}}\sigma_{m}H_{z}dS_{4},$$

where *S*_4_ is the magnetic pole surface (N pole of the movable magnet ring) with the surface magnetic charge density of *σ_m_*. The total *z*-component magnetic force *F_total_* on the movable part (including the pedal, the movable plate, the impact block, and the movable magnet rings) is given by

(5) $$F_{total}=2F_{z}.$$

The magnetic force *F_total_* makes the movable part return to its original position under human foot strikes, and it can be expressed as a power series of the displacement *z* that is given by

(6) $$F_{total}=k_{1}z+k_{2}z^{2}+k_{3}z^{3},$$

where *k_i_* (*i* = 1, 2, 3) is the coefficient of the polynomial.

**Figure 3:**
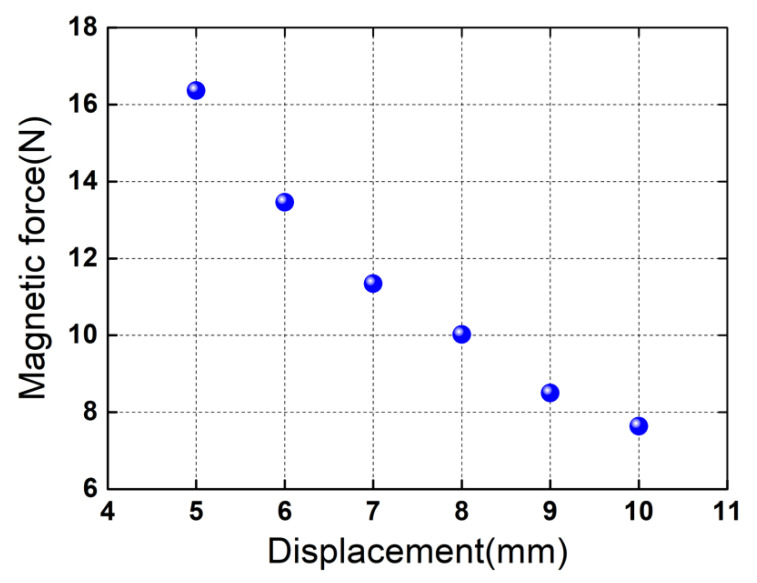
FEA predictions of the total *Z*-component magnetic force on the movable part as a function of the displacement *Z*. The blue spherical symbol denotes the total *Z*-component magnetic force for the corresponding displacement. The origin of coordinate is established at the geometric center of the top surface of the fixed magnet ring. The total *Z*-component magnetic force is obtained based on the force superposition of the two magnetic springs.

Maxwell 3D was used to analyze the magnetic force *F_total_* on the movable part as a function of the displacement *Z*. Here, *Z* is the displacement of the movable magnet ring, which also represents the distance between the top surface of the fixed magnet ring and the bottom surface of the movable magnet ring. In simulation, one magnetic spring is modeled, and 3D transient field analysis is conducted. The movable magnet ring moves along the *Z*-axis, while the fixed magnet ring is static. The material NdFe35 in the software is selected for the magnet rings. The default boundary conditions and the automatic meshing are employed. The FEA simulation results are plotted in Figure 3. As can be seen from Figure 3, the magnetic force exhibits nonlinear properties, and the coefficients in Equation (6) can be obtained using the polynomial fitting method.

**Figure 4:**
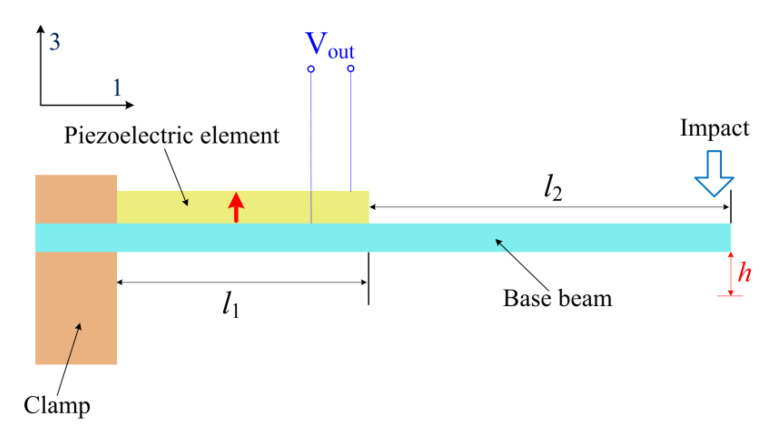
Schematic diagram of the piezoelectric unimorph cantilever under impact. The red arrow denotes the polarization direction of the piezoelectric element.

Considering one piezoelectric unimorph cantilever shown in Figure 4, the cantilever has a length ratio of *a* > 1(nonpiezoelectric base beam to piezoelectric element). Under the impact of the impact block resulting from the foot strikes, the following piezoelectric constitutive equations for the piezoelectric element can be applied:

(7) $$S_{1}=s_{11}^{E}T_{1}+d_{31}E_{3},$$

(8) $$D_{3}=d_{31}T_{1}+\varepsilon_{33}^{T}E_{3},$$

where *S*_1_ and *T*_1_ are the strain and stress, respectively, $s_{11}^{E}$ denotes the piezoelectric elastic compliance, *d*_31_ is the piezoelectric coefficient, *E*_3_ and *D*_3_ are, respectively, the electric field and electric displacement along the polarization direction in Figure 4 (namely the thickness direction of the piezoelectric element), and $\varepsilon_{33}^{T}$ represents the permittivity at constant stress. Assuming that the Young’s modulus of the base beam is much less than that of the piezoelectric element (*E_p_*/*E_s_* > > 1), the stress is then given by [26,27]

(9) $$T_{1}=\frac{kE_{p}t_{p}h}{8E_{0}b}(l_{1}+2l_{2}),$$

where *k* is the effective spring constant of the entire cantilever (namely the cantilever with the length of *l* = *l*_1_ + *l*_2_ in Figure 4), *E_p_* is the Young’s modulus of the piezoelectric element, *t_p_* is the thickness of the piezoelectric element, *h* is the tip displacement of the cantilever, *l*_1_ is the length of the piezoelectric element, *l* = *l*_1_ + *l*_2_ is the length of the entire cantilever, *b* is the width of the cantilever, and *E*_0_ is the bending modulus per unit width for the composite part (including the base beam and the piezoelectric element) with the length of *l*_1_, which is given by

(10) $$E_{0}=\frac{E_{p}t_{p}^{3}}{12}+\frac{E_{s}t_{s}}{6}(2t_{s}^{2}+2t_{p}^{2}+3t_{s}t_{p}),$$

where *E_s_* and *t_s_* are, respectively, the Young’s modulus and the thickness of the base beam. The electric displacement and the electric field can be respectively expressed as

(11) $$D_{3}=\frac{Q}{A},$$

(12) $$E_{3}=\frac{V}{t_{p}},$$

where *Q* and *V* are the induced charge and voltage, respectively, and *A* is the area of the electrode. When a load resistance *R_L_* is connected to the electrodes of the piezoelectric element, the generated current and the load voltage can be respectively expressed as

(13) $$I(t)=\frac{dQ}{dt}=\frac{Ad_{31}kE_{p}t_{p}(l_{1}+2l_{2})}{8E_{0}b}\frac{dh}{dt}+\frac{A\varepsilon_{33}^{T}}{t_{p}}\frac{dV}{dt},$$

(14) $$V(t)=R_{L}I(t)=\frac{R_{L}Ad_{31}kE_{p}t_{p}(l_{1}+2l_{2})}{8E_{0}b}\frac{dh}{dt}+\frac{R_{L}A\varepsilon_{33}^{T}}{t_{p}}\frac{dV}{dt}.$$

Supposing that the gaits are all the same during human walking (running) with a period of *t*_0_, the RMS of *V*(*t*) can then be calculated by

(15) $$V_{RMS}=\sqrt{\frac{1}{t_{0}}\int_{0}^{t_{0}}V^{2}(t)dt},$$

The RMS power delivered to *R_L_* is given by

(16) $$P=\frac{V_{RMS}^{2}}{R_{L}}=\frac{\int_{0}^{t_{0}}V^{2}(t)dt}{R_{L}t_{0}}.$$

Based on Equations (14) and (16), the generated power depends on the tip displacement of the base beam after determining the materials and the structural parameters of the piezoelectric unimorph cantilever. As the force on the pedal resulting from human foot strikes is much greater than the magnetic force of the magnetic springs, the maximal tip displacement is dependent on the initial distance between the stopper and the movable magnet ring and the initial separating distance between the impact block and the base beam.

## 3. Results and Discussions

**Figure 5:**
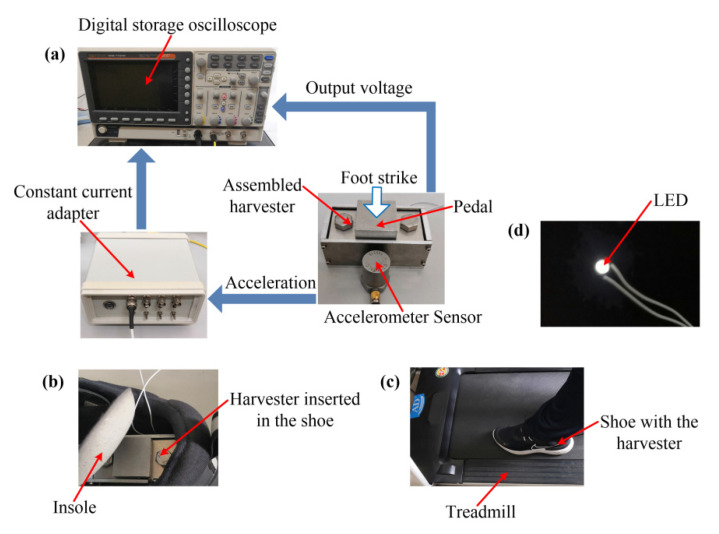
Experimental setup: (**a**) measuring principle of the induced output voltages and the accelerations; (**b**) assembled harvester inserted into a shoe; (**c**) tester wearing the harvester walking (running) on a treadmill; (**d**) LED lighted up by the harvester at 3 km/h.

Experiments were carried out using an assembled prototype with a maximal dimension of 80 mm × 40 mm × 42 mm (the dimension of the pedal is 30 mm × 32 mm × 10 mm) to investigate the output performances of the piezoelectric heterostructure. The piezoelectric elements (PZT5H) of the two piezoelectric unimorph cantilevers are connected in parallel. The assembled harvester was inserted into a shoe. The foot strikes resulted from the realistic human motion (walking at 3–6 km/h and running at 7 km/h) of a tester (with a weight of 66.5 kg) waking (running) on a treadmill. Figure 5 shows the experimental setup of the energy-harvesting system. An accelerometer sensor (CT1100LC) was used to conduct the measurements of the accelerations normal to the swing direction of the foot. Stable operation voltages were provided by a constant current adapter (CT5204) for the accelerometer sensor. The generated voltages were measured with a digital storage oscilloscope (GDS-1104R).

**Figure 6:**
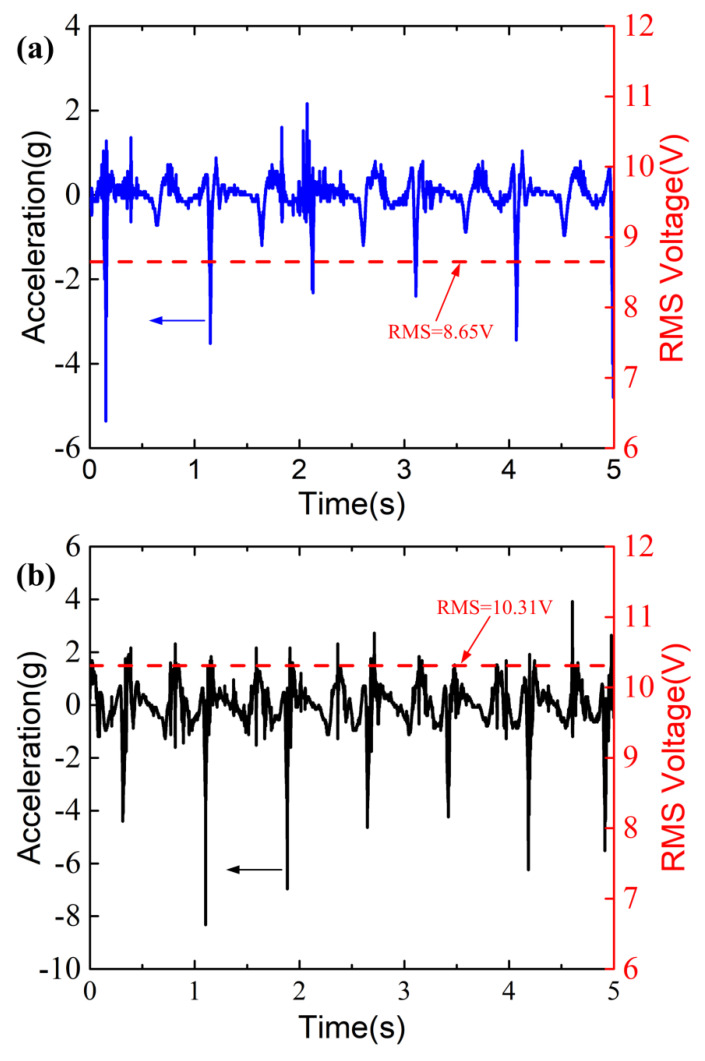
Acceleration resulting from the foot strikes and the corresponding RMS voltage for (**a**) 3 km/h and (**b**) 6 km/h. The blue and black arrows indicate that the left labels of the figures apply to the waveforms of the accelerations. The red arrows point to the RMS voltages plotted by the red dash lines.

Figure 6 plots the acceleration waveforms and the induced open-circuit RMS voltages at different walking speeds. The initial separating distance between the top surface of the stopper and the bottom surface of the movable magnet ring is *d* = 5 mm. As can be seen in Figure 6, the acceleration waveforms exhibit multiple peaks due to the multiple foot strikes in the given interval of 0–5 s. The peak absolute value of the acceleration exceeds 5 g at 3 km/h and that exceeds 8 g at 6 km/h in the interval of 0–5 s. The RMS of the acceleration is 0.4056 g at the walking speed of 3 km/h (the corresponding walking frequency is about 1 Hz), and the RMS acceleration increases to 0.7594 g when the walking speed is increased to 6 km/h (the corresponding walking frequency increases to about 1.27 Hz). The RMS of the acceleration at 6 km/h is greater than that at 3 km/h due to the stronger foot strikes. The induced RMS voltage is 8.65 V at 3 km/h, and the voltage increases to 10.31 V at the faster walking speed of 6 km/h. Here, the RMS values of the accelerations and the induced voltages are calculated using the sample points in the interval of 0–5 s, which are given by

(17) $$a_{RMS}=\sqrt{\frac{1}{k_{a}}\sum_{i=1}^{k_{a}}a_{i}^{2}},$$

(18) $$V_{RMS}=\sqrt{\frac{1}{k_{V}}\sum_{i=1}^{k_{V}}V_{i}^{2}},$$

where *k_a_*is the total sample points of the acceleration (*k_a_* *=* 10,000), *k_V_*is the total sample points of the voltages, *k_a_ = k_V_*, and *a_i_* and *V_i_* are, respectively, the acceleration and the voltage of the given sample point *i*.

**Figure 7:**
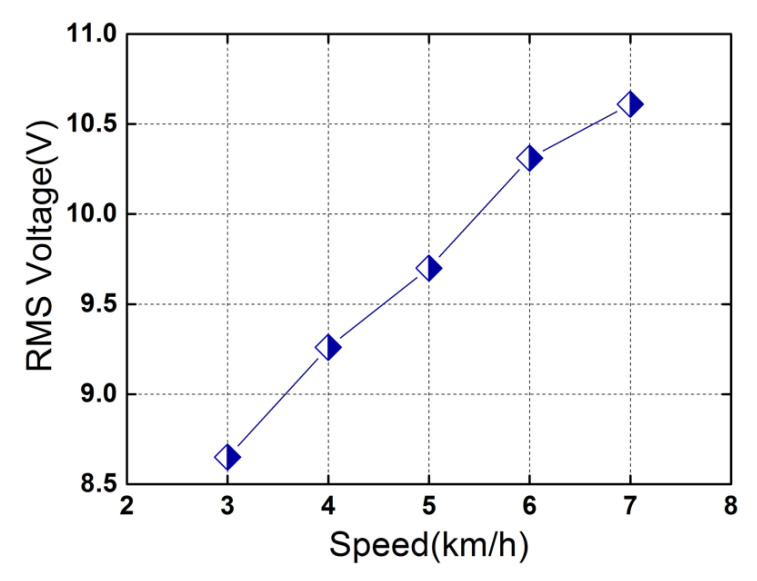
Induced open-circuit RMS voltage as a function of the motion speed. The symbols denote the voltages at different speeds.

Figure 7 shows the induced open-circuit RMS voltage of the piezoelectric heterostructure at different human walking (running) speeds. The initial separating distance *d* = 5 mm. It can be seen from Figure 7 that the induced RMS voltage shows a monotonous increase from 8.65 V to 10.61 V when the speed increases from 3 km/h to 7 km/h. The monotonous increase of the voltage is attributed to the increasing acceleration and striking frequency when the speed increases. It should be noted that the induced RMS voltages might be different for different testers as gaits, heights, and weights vary from person to person.

**Figure 8:**
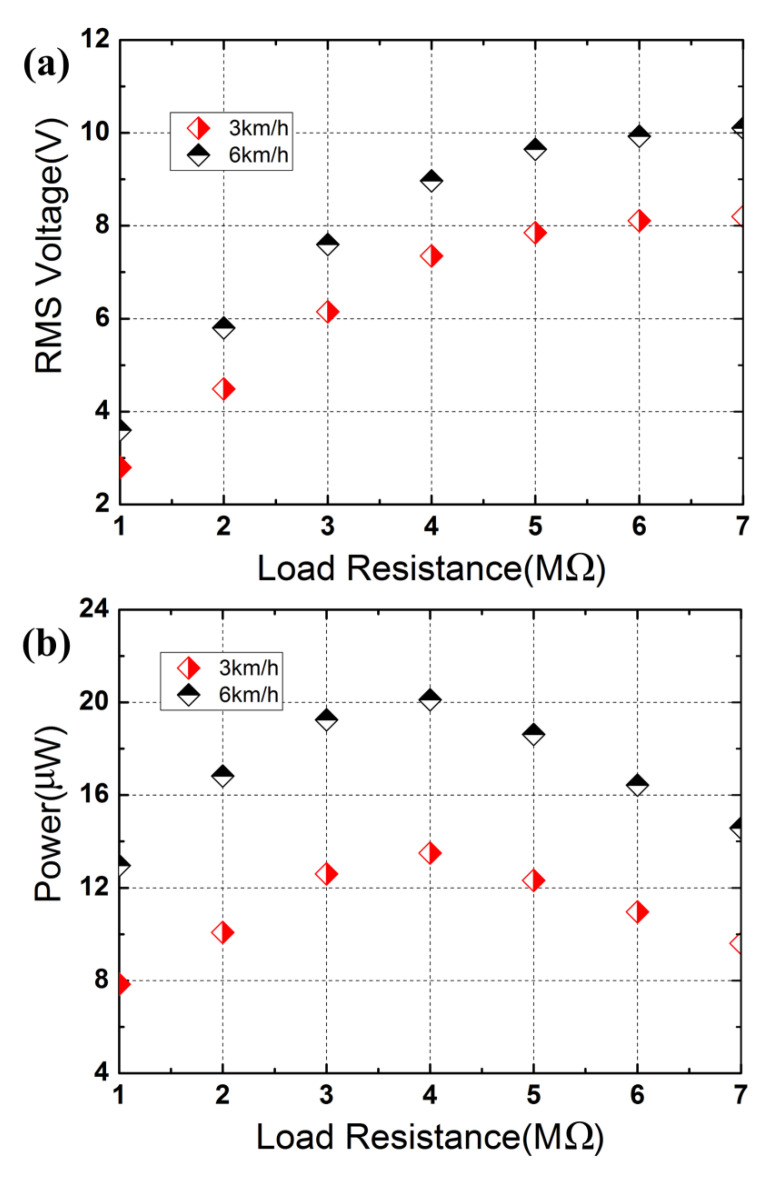
Load voltage and average power as a function of the load resistance: (**a**) RMS voltage; (**b**) average power.

Figure 8 plots the RMS voltage and the corresponding average power as a function of load resistance *R_L_* under human foot strikes. As can be seen in Figure 8a, the load voltage increases with the load resistance. The voltage increases from 2.8 V to 8.2 V when the load resistance is increased from 1 MΩ to 7 MΩ (Δ*R_L_* = 1 MΩ) at 3 km/h, while the voltage rises from 3.6 V (across 1 MΩ resistive load) to 10.1 V (across 7 MΩ resistive load) at a higher walking speed of 6 km/h. The load voltages across the 7 MΩ resistive load (8.2 V for 3 km/h and 10.1 V for 6 km/h) are close to the open-circuit voltages (8.65 V for 3 km/h and 10.31 V for 6 km/h, respectively). The average powers are calculated based on the following equation:

(19) $$\bar{P}=\frac{V_{RMS}^{2}}{R_{L}},$$

where *V_RMS_* is the load RMS voltage across load resistance *R_L_*. The average powers are calculated based on the experimental results in Figure 8a, which are plotted in Figure 8b. From Figure 8b, it can be seen that the average power first increases with the load resistance and then decreases with further increases in the load resistance. The maximal average power in two conditions (3 km/h and 6 km/h) is, respectively 13.506 μW and 20.115 μW (the load voltage across the matching load resistance of 4 MΩ is 7.35 V at 3 km/h and that at 6 km/h increases to 8.97 V). The maximal average power at 6 km/h is ~1.49 times larger than that at 3 km/h, indicating that more mechanical energy resulting from foot strikes can be harvested at the higher speed of 6 km/h.

**Figure 9:**
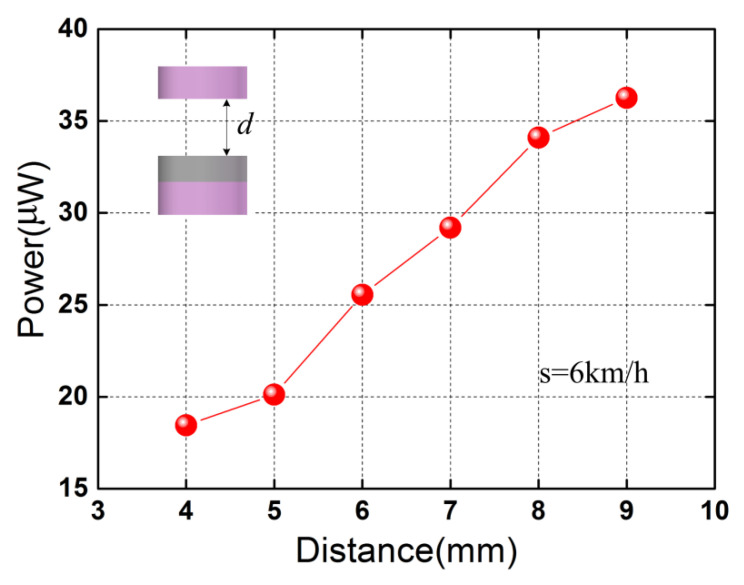
Maximum average power versus initial separating distance *d* at the walking speed of 6 km/h. The red spherical symbols denote the maximum average powers for different initial separating distances.

The tip displacement of the base beam is a key parameter for power generation that is relevant to the initial separating distance between the bottom surface of the movable magnet ring and the top surface of the fixed magnet ring *d_m_* (*d* = *d_m_* − *d*_s,_ *d*_s_ is the height of the stopper). Figure 9 shows the generated maximum average power (across the matching load resistance) for different initial separating distances at 6 km/h. In Figure 9, the initial distance between the impact block and the base beam is kept the same by using impact blocks of different sizes. It can be seen in Figure 9 that the maximum average power increases with the initial separating distance, which rises from 18.45 μW to 36.26 μW as the distance *d* is increased from 4 mm to 9 mm. The monotonous increase with the initial separating distance *d* in Figure 9 is attributed to the increasing tip displacement. Greater maximum average power can be generated using the piezoelectric elements with a higher *d*_31_ (e.g., piezoelectric single crystal PMN-PT), increasing the number of the piezoelectric unimorph cantilevers and adopting the *d*_15_ working mode of the piezoelectric elements.

**Figure 10:**
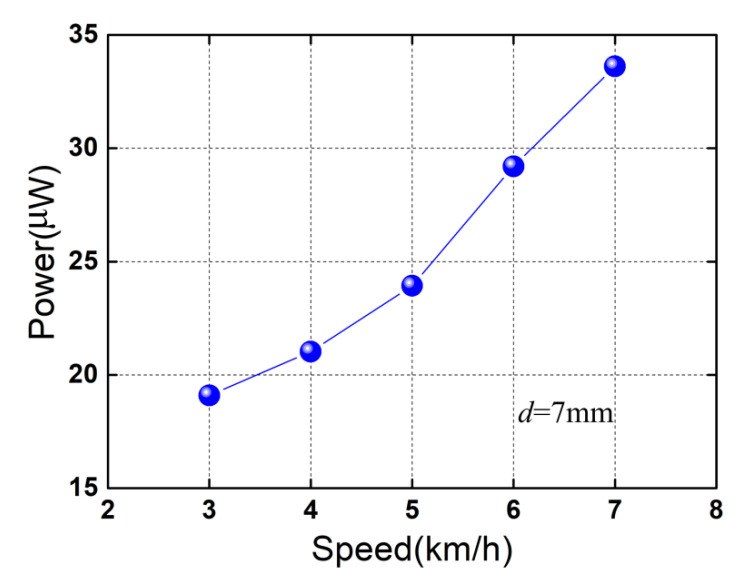
Maximum average power as a function of the human motion speed. The blue spherical symbols denote the maximum average powers for different speeds.

Figure 10 plots the maximum average power at different motion speeds when *d* = 7 mm. As expected, the maximum average power increases with the motion speed, which increases from 19.1 μW to 33.61 μW as the speed is increased from 3 km/h to 7 km/h. By considering the instantaneous powers, the peak power reaches 1586 μW at 7 km/h (*d* = 7 mm), and the corresponding power density is ~11.8 μW/cm^3^ (peak) when the maximum occupied volume of the device is considered and is ~13.767 mW/cm^3^ (peak) when only the volumes of piezoelectric elements are considered. The output performances of the devices for energy harvesting from human foot strikes are listed in Table 1. The feasibility of the proposed device with the merits of the magnetic springs is validated for harvesting mechanical energy from foot strikes. Several design implications of the piezoelectric heterostructure can be summarized to improve the output powers: (1) The RMS voltage increases with the piezoelectric constant. Therefore, higher output voltages can be obtained by using piezoelectric materials with a higher *d*_31_, such as piezoelectric single crystal PMN-PT. (2) For the total capacitance of the heterostructure, *C_t_* = *nC* (when connected in parallel). Here, *n* is the number of piezoelectric unimorph cantilevers, and *C* is the capacitance of each piezoelectric element. More output power can be obtained by increasing the number of piezoelectric unimorph cantilevers. (3) The working mode of the piezoelectric elements is also an important factor for power generation. More output power can be obtained when adopting the *d*_15_ working mode, which can be realized by polarizing the piezoelectric element along the length [28,29]. (4) The maximum tip displacement of the piezoelectric unimorph cantilever is relevant to the initial separating distance *d*. It is feasible to increase *d* to achieve large tip displacement of the piezoelectric unimorph cantilever (within the allowed impact load) for each gait cycle.

## 4. Conclusions

In summary, a piezoelectric heterostructure employing magnetic springs for harvesting mechanical energy from human foot strikes is presented. The proposed device is based on the impact of a moving block on the tips of the piezoelectric unimorph cantilevers. The piezoelectric elements of the cantilevers then deform in the *d*_31_ mode, which generate voltage output. The use of the magnetic springs makes the device suitable for high-amplitude movements induced by human foot strikes. Two piezoelectric unimorph cantilevers were adopted to enhance the total capacitance of the heterostructure. The working principle of the heterostructure was stated, and the feasibility under human foot strikes was validated. A prototype of the harvester was characterized experimentally. The experimental results show that the induced open-circuit RMS voltage exhibits a monotonous increase with the walking (running) speed. An average power of 20.115 μW is generated across a matching load resistance of 4 MΩ with an initial separating distance of 5 mm at the walking speed of 6 km/h. Further optimization of the impact harvester is possible. The output average power can be improved by optimizing the materials, the structural parameters, and the working mode of the piezoelectric elements.

## Figures and Tables

**Table 1:** Comparison of devices for energy harvesting from human foot strikes.

Reference	Mechanism	Size	Power or Power Density
Hou et al. [21]	triboelectric	27 × 9 × 0.3 cm^3^	1.4 mW (maximum)
Haque et al. [22]	triboelectric	133 × 0.2 cm^3^	250 μW
Xie et al. [23]	piezoelectric	-	0.41 mW/cm^3^
Zhao et al. [24]	piezoelectric	8 × 5 cm^2^ (area of PVDF)	~1000 μW
Fan et al. [25]	piezoelectric	4.5 × 3 × 2.4 cm^3^	0.35 mW
Proposed	piezoelectric	8 × 4 × 4.2 cm^3^	1586 μW (peak) and 33.61 μW (average)

## Data Availability

The data used to support the findings of this study are included within the article.
